# Functional Quality and Microbiological Stability of Grape Juice During Processing by UV-C Light Compared to Mild Heat Treatment and Evolution of the Parameters During Cold Storage

**DOI:** 10.3390/foods14122056

**Published:** 2025-06-11

**Authors:** Cielo Char, Carla Vegas, Nalda Romero, Luis Puente-Diaz, Jaime Ortiz-Viedma, Marcos Flores

**Affiliations:** 1 Departamento de Ciencias de los Alimentos y Tecnología Química, Facultad de Ciencias Químicas y Farmacéuticas, Universidad de Chile, Av. Dr. Carlos Lorca Tobar 964, Independencia, Santiago P.O. Box 1004, Chile; carlav.vegas@gmail.com (C.V.); nromero@uchile.cl (N.R.); lpuente@ciq.uchile.cl (L.P.-D.); jaortiz@uchile.cl (J.O.-V.); 2 Departamento de Horticultura, Facultad de Ciencias Agrarias, Universidad de Talca, Campus Lircay, Talca 3460000, Chile; marcos.flores@utalca.cl

**Keywords:** grape juice, ultraviolet light, food safety, antioxidants, polyphenols, minimal processing, shelf life

## Abstract

The effect of UV-C was assessed on microbiological stability, functional compounds, and quality factors of white grape juice compared to mild thermal treatment (MTT). The inactivation kinetics of *Escherichia coli* (target microorganism) and *Saccharomyces cerevisiae* in grape juice by UV-C and MTT (75 °C) were evaluated to define the processing time. The comparative effect on the functional compounds was evaluated. Additionally, microbiological stability and quality factors were assessed during storage at 4 °C. The grape juice was characterized (pH 3.7, 25 °Brix, absorption coefficient 44.2 cm^−1^, total phenols (TP) 214 mg GAE/L). UV-C (569 mJ/cm^2^) reduced *E. coli* to 5.03 log CFU/mL in 12.5 min, preserved TP (235 mg GAE/L), and antioxidant capacity (AC 4.5 mM TE/L). Thermal treatment reduced *E. coli* to 5.14 log CFU/mL in 180 s, increased TP (237–312 mg GAE/L), and maintained the AC of the juice. During storage, UV-C and MTT controlled microbiota growth, extending the time of microbiological stability by 42%. All the treatments showed a similar gradual loss of TP during storage. However, MTT has better-preserved color parameters. In conclusion, UV-C was effective from a microbiological perspective without compromising the functional quality of juice. However, further research is needed to improve color.

## 1. Introduction

Fruit juices are considered practical, convenient, and nutritious foods, and their consumption has increased in recent years [1]. The white grape *Vitis vinifera* (L.) cv. Thompson Seedless is one of the most widely cultivated seedless grape cultivars in the world and is available over a wide time during the year. This variety has been used for fresh markets, raisins, and for the production of juices, concentrates, and wines [2]. This white grape variety is well-suited for processing due to its minimal color development, low pulp level, ease of pressing, neutral taste, and high yield. It has also been used as a sweetener for juices and foods due to its high concentration of soluble solids (>20 °Brix) [3].

Grapes are rich in phenolic compounds such as flavonoids quercetin, rutin, and kaempferol; anthocyanins such as procyanidin; flavan-3-ols such as catechin and epicatechin; non-flavonoids such as resveratrol; and phenolic acids such as benzoic and cinnamic acids [4]. These functional compounds have antioxidant properties that protect against oxidative stress, generating positive health effects, including antimicrobial, antioxidant, anti-inflammatory, antitumor, vasodilator, and antihyperglycemic activities [5,6].

The microorganisms particularly associated with unpasteurized grape juice spoilage are yeasts commonly found on grape skins, such as *Kloeckera apiculata* and *Candida* spp., which play a role in the early stages of grape juice fermentation, while *Saccharomyces cerevisiae* dominates the fermentation and gas formation in grape juice [7]. The osmophilic yeasts *Zygosaccharomyces rouxii* and *Schizosaccharomyces pombe* are most frequently isolated from grape juice concentrate [8]. Lactic acid and acetic acid bacteria (e.g., *Gluconobacter oxydans*) also cause spoilage in unpasteurized fresh juices, and the spore-forming bacterium *Alicyclobacillus acidoterrestris* in pasteurized juices due to its thermal resistance [9,10].

Even though fruit juices are usually low-risk microbiological foods due to their acidic pH, they can also be vehicles for pathogenic bacteria that cause foodborne illnesses. In this regard, 25 outbreaks of foodborne disease associated with fruit juices were reported worldwide during the period 2005–2019 [11]. The juices involved in the highest number of outbreaks were apple juice or cider (16 outbreaks), orange juice (2 outbreaks), and, to a lesser extent, açai, guava, carrot, mamey, and juice mixes. Of these outbreaks, 11 were caused by *Escherichia coli*, most of them by the Shiga toxin-producing serotype *E. coli* O157:H7; 4 outbreaks were caused by *Salmonella spp.*; 4 by *Cryptosporidium parvum; 3* by *Trypanosoma cruzi*, and 1 each by *Clostridium botulinum*, *Campylobacter jejuni*, and Hepatitis A virus [11]. Additionally, grapes have been reported to cause an outbreak of *Escherichia coli* O157:H7 [12].

Although thermal treatments are very effective for microbial inactivation, they often cause a loss of organoleptic (texture, flavor, and color changes) and nutritional quality (loss of vitamins and bioactive compounds) [13,14]. In this sense, it has been reported that thermal pasteurization (97 °C for 60 s) of white grape juice significantly affected the color (ΔE), decreased the content of total phenols, anthocyanins, and antioxidant capacity during storage, and reduced the overall juice acceptance with a decrease in aroma, sweetness and acidity scores, and an increase in bitterness scores, compared to fresh juice and juice treated by non-thermal technologies [15]. For this reason, non-thermal processing technologies for cold pasteurization of liquid foods have emerged, such as short-wave ultraviolet light (UV-C) [16,17]. The UV-C light (λ: 253.7 nm) is effective in inactivating both spoilage microorganisms, such as fungi and yeasts, as well as pathogenic bacteria [13,16,17]. It is a low-cost, energy-efficient, and versatile technology. The process is simple, preserves the texture and nutrients of food, and does not leave toxic chemical residues, so it is considered a clean technology [17,18].

The impact of UV-C light on the total phenol (TP) content and antioxidant capacity (AC) of fruit juices shows variable results depending on the specific food matrix and the dose applied, according to the existing literature. Studies on mango juice indicate that exposing it to UV-C light increased TP and AC by 31% and 12%, respectively [18]. Similarly, research on pear juice found that phenolic compounds were increased by 9% [19]. However, studies involving orange juice and carrot juice reported that both TP and AC values remained unchanged after UV-C treatment [20,21]. Conversely, some researchers observed an 11% decrease in antioxidant capacity in apple juice [22]. Overall, these findings highlight the need for further studies to address these knowledge gaps.

Many characteristics influence the efficacy of UV-C, such as the fruit juice matrices (e.g., UV absorbance, turbidity, soluble solid content, pH, color, etc.); the target microorganism (e.g., type of microorganism/strain, acid adaptation, physiological state, single/composite inoculum, spore production, etc.); and the process parameters including UV-C light source and reactor conditions (e.g., continuous/batch, thickness, volume, flow rate, residence time, homogenization conditions and cleaning capability) [1,16,23]. These challenges limit the application of UV-C light to clear, liquid foods with no suspended solids or pigments and low viscosity. This demonstrates the need to investigate and address the existing challenges to develop the immense potential of UV-C light for the technology to be applicable to a broader range of products on an industrial scale, since, so far, it is restricted to the treatment of a few products, such as apple cider.

Based on the above, this study aims to address the gaps identified in applying UV-C light to cloudy juices, such as cold-pressed grape juice that contains green pigments, suspended solids, high levels of soluble solids, and a high absorption coefficient. The research will examine the effects of UV-C treatment on functional compounds, microbiological stability, and other quality factors, and will compare these effects to those of mild thermal treatment (MTT). For this purpose, the inactivation kinetics of *E. coli* (target microorganism involved in juice outbreaks) and *S. cerevisiae* (highly UV-C resistant spoilage yeast) were evaluated by both UV-C and MTT at 75 °C to define the processing time that would achieve a 5-log reduction of the relevant microorganism. The defined treatments were then applied to uninoculated juice to evaluate the comparative effect of both minimal processes on the functional quality of the juice. Additionally, microbiological stability, functional compounds, and quality factors were evaluated during storage at 4 °C for 21 days.

## 2. Materials and Methods

### 2.1. Materials, Reagents, and Culture Media

White grape (*Vitis vinifera* L.) cv. Thompson Seedless was purchased from the local market in Santiago, Chile. Reagents such as the radicals 2,2′-azobis(2-amidinopropane) dihydrochloride (AAPH·) and 2,2-diphenyl-1-picrylhydrazyl (DPPH), Trolox (T), fluorescein, sodium chloride, potassium chloride, disodium hydrogen phosphate, potassium dihydrogen phosphate, sodium hydroxide, hydrochloric acid 10%, gallic acid (GA) were purchased from Merck (Darmstadt, Germany). Folin–Ciocalteu reagent and methanol p.a. were purchased from Winkler (Santiago, Chile). Culture media such as tryptone soy agar (TSA), potato dextrose agar (PDA), plate count agar (PCA), chloramphenicol agar (YGC), tryptic soy broth (TSB), and 2% glucose Sabouraud broth were also purchased from Merck (Darmstadt, Germany). Petrifilm™ plates for coliforms/*E. coli* were purchased from 3M™ (Two Harbors, MN, USA).

### 2.2. Extraction of Grape Juice

Freshly harvested Thompson Seedless grapes (20 kg) (March 2024 harvest) with excellent organoleptic appearance were purchased from the local market in Santiago, Chile. They were preselected, eliminating those with visual defects, and washed by immersion in water. They were rinsed by changing the water twice, and the excess water was drained through a plastic mesh net. A sanitized domestic cold presser (Easyways Slow Juicer, Pro D122, Santiago, Chile) was used to extract the juice. The whole grapes were processed through the auger, which extracted the juice, separating it from the skin and seeds, which were discarded. The juice then passed through the filter and was collected in the tank. Each time the juicer’s tank reached its maximum capacity (1.5 L), the juice was immediately aliquoted by transferring 700 mL to sterile glass bottles, and it was frozen at −18 °C until use.

### 2.3. Characterization of Grape Juice

Grape juice was characterized by the following determinations: pH with a pH meter (Jenco Instruments, 6177pH/ORP, San Diego, CA, USA); soluble solids content was measured with a digital refractometer (RX-1000 Atago, Atago Co, Carnation, WA, USA); titratable acidity was determined by titration with a 0.1 M NaOH and the results were expressed in g/100 mL of the primary organic acid (tartaric acid) according to the AOAC methodology [24].

To measure the optical properties of the juice, such as the absorption coefficient (α) of UV-C light, the absorbance of the sample was measured using a UV/visible spectrophotometer (Genesys™ 10S Bio, Thermo Scientific, Waltham, MA, USA) at a wavelength of 254 nm [16]. Different dilutions of the juice were measured in a 1 cm thick quartz cuvette, and the α coefficient was calculated with Equation (1):

(1) $$\alpha =2.303\;\cdot A\;\cdot d^{-1}$$

where the α coefficient is defined as the absorbance (*A*) divided by the optical path length *d* (cm^−1^) [25].

The color of the samples was measured before and after the treatments, and the color variation over time was calculated. Color coordinates in the CIElab space, L*: luminosity; a*: red/green coordinates; b*: yellow/blue coordinates were measured with a spectrocolorimeter (Hunter Associates Laboratory, Reston, VA, USA). The colorimeter was standardized with black and white standards before each measurement, and 30 mL of each sample was used in an optically clear glass cell. The overall color difference ∆*E* was determined through Equation (2).

(2) $$∆E=\sqrt{{\left(∆L^{*}\right)}^{2}+{\left(∆a^{*}\right)}^{2}+{\left(∆b^{*}\right)}^{2}}$$

Total polyphenols were determined by spectrophotometry based on the Folin–Ciocalteu colorimetric oxidation–reduction reaction [26]. The absorbance of the samples was measured at a wavelength of 765 nm, against a blank. The TP content was expressed in gallic acid equivalents (mg GAE/L) [27].

In addition, the antioxidant capacity of the juice was determined using two methodologies: (a) oxygen radical absorption (ORAC) following the methodology of Fuentes et al. [28] using a fluorimeter (Biotek FLx800, Agilent BioTec, Santa Clara, CA, USA), and the results were expressed as Trolox equivalents (mM TE/L); (b) DPPH^·^ radical following the methodology of Brand-Williams et al. [29]. The radical decolorization was determined at 517 nm, its concentration was quantified using Trolox as a standard, and the results were expressed as Trolox equivalents (mM TE/L).

### 2.4. Microbial Inactivation Kinetics

#### 2.4.1. Inoculum Preparation

The inoculum of *Escherichia coli* ATCC 35,218 was prepared by reactivating material from a frozen stock culture by transferring a loopful to a bottle with 30 mL of trypticase soy broth (TSB) plus 0.6% *w*/*v* yeast extract (YE) and incubating at 37 °C under shaking for 24 h. Then, trypticase soy agar (TSA) slants were made, incubated under the same conditions, and stored at 4 °C in airtight containers until use. Before each run, the inoculum was prepared by transferring material from a strip to a bottle containing 30 mL of TSB, which was incubated under the same conditions for 24 h until the stationary phase was reached. The final inoculum concentration was ≈5.0 × 10^8^ CFU/mL.

The inoculum of *Saccharomyces cerevisiae* ATCC 9763 was prepared by transferring material from a frozen stock culture (−18 °C) to a bottle with 30 mL of Sabouraud glucose 2% (SG) broth and incubating at 25 °C with shaking for 48 h. Potato dextrose agar (PDA) slants were then plated, incubated under the same conditions, and stored at 4 °C in airtight containers until use. Before each run, the inoculum was prepared by transferring material from a strip to a bottle containing 30 mL of SG, which was incubated under the same conditions for 48 h until the stationary phase was reached. The final inoculum concentration was ≈6.0 × 10^7^ CFU/mL.

#### 2.4.2. Application of UV-C Light Treatments

A continuous flow UV-C system consisting of 2 UV-C lamps (TUV36W, Philips, Warsaw, Poland; 36W, length 120 cm) connected in series, with a monochromatic emission peak at 254 nm was used. Each lamp operated as a thin film flow reactor with a 0.3 cm annular gap between the lamp and the outer glass cover, through which the juice was pumped by a peristaltic pump (Masterflex Easy-load 7518-00, Cole Palmer, Vernon Hills, IL, USA). Therefore, the juice was in direct contact with the external surface of the UV-C lamp, along its entire length.

The juice (700 mL) was placed in the feed tank for each treatment and inoculated with yeast or bacterial culture, reaching initial concentrations of ≈1–5 × 10^6^ CFU/mL. It was continuously homogenized using a magnetic stirrer (IKA, Königswinter, Germany), and the temperature was maintained with an ice bath. The juice was immediately recirculated through the equipment at a flow rate of 6 mL/s. The samples were taken at various UV-C exposure times, up to a maximum of 20 min, which was estimated to achieve a reduction of 5 logarithmic cycles of the microorganisms. The samples were placed in an ice bath until analysis. Uninoculated juice was used to determine functional compounds and analyze storage stability. Each complete test was conducted in triplicate.

Before and after the treatments, the UV-C lamps were sanitized by continuous recirculation of water with neutral detergent CIP for 10 min, then rinsed with sterile water for 10 min. Finally, the lamps were turned on for 15 min to stabilize the emission and disinfect the irradiation area. At the end of the treatment, the system was washed with detergent, rinsed with water, sanitized with a 5% (*v*/*v*) chlorine solution for 10 min, and rinsed twice with distilled water for 10 min.

#### 2.4.3. Determination of the Delivered UV-C Dose

The germicidal or delivered dose (mJ/cm^2^), which represents explicitly the energy delivered to the microorganisms causing inactivation (excluding the energy absorbed or dispersed by the fluid constituents), was determined by an actinometric reaction [30]. For this purpose, the iodide/iodate chemical actinometer was used following the methodology by Rahn [30], which was adapted for a continuous flow UV-C system. Solutions of 0.6 M potassium iodide and 0.1 M potassium iodate in 0.01 M phosphate buffer adjusted to pH 9.25 were used. These solutions were freshly prepared, mixed, and irradiated. The photons absorbed by the iodide/iodate chemical actinometer from the irradiated solution caused the formation of triiodide, which was quantified by measuring the absorbance of the sample at 352 nm. The maximum delivered UV-C dose was 900 mJ/cm^2^ for 20 min treatments.

#### 2.4.4. Application of Mild Thermal Treatment (MTT)

The mild temperature-long time thermal treatment was performed at 75 °C using a three-neck round-bottom flask placed on a heating mantle. The temperature was measured in one neck with a thermocouple connected to a recorder, the second neck was for the entry of the sample and the inoculum of the microorganism to be studied, and a thermometer was introduced in the third neck. The round-bottom flask was placed approximately 3 cm from the heating mantle, leaving space for no direct contact between the flask and the mantle. Under the mantle was placed a magnetic stirrer (Heidolph Instruments, Schwabach, Germany) that kept the sample homogenized. The temperature of the system was calibrated before each use with distilled water. For each treatment, 99 mL of juice was used. Once the juice reached the desired temperature (75 °C), it was inoculated with 1 mL of the microorganism of interest and immediately the sample corresponding to time = 0 was taken (N_0_ for *E. coli* was ≈ 1–4 × 10^6^ CFU/mL; N_0_ for *S. cerevisiae* was ≈ 1–2 × 10^6^ CFU/mL). The rest of the kinetic samples were subsequently taken at times 30, 60, 90, 120, and 180 s and placed in an ice bath for microbiological analysis. Uninoculated juice was used to determine functional compounds following the same procedure. Each complete assay was performed at least in triplicate.

#### 2.4.5. Enumeration of Microorganisms

For each treatment, survivor counts were determined by the plate count method. Two successive dilutions were plated in duplicate, and 0.1 mL of the corresponding dilution was spread on the plate surface (DL: 100 CFU/mL), but when higher UV-C doses were applied, 0.1 mL of the undiluted juice was used (DL: 10 CFU/mL). Each complete assay was conducted at least in triplicate (*n* = 6). For *E. coli*, TSA+ 0.6% YE agar was used and incubated for 24 h at 37 °C. In the case of *S. cerevisiae*, it was plated on PDA agar and incubated for 48 h at 25°C. The data obtained were used to plot the survival curves as a function of time [log N/N_0_ vs. time] (where N is the concentration of microorganisms at a given time (CFU/mL), and N_0_ is the initial microbial concentration).

### 2.5. Storage Stability

Based on the results of the previous tests, the processing time for each treatment was selected, which was the time at which 5 log reductions of *E. coli* (relevant microorganism) were achieved. UV-C and thermal treatments (75 °C) were applied to uninoculated juice for the selected times (12.5 min and 180 s, respectively) following the procedures described in Section 2.4. In addition, one untreated control was included. The processed juice samples were distributed in 100 mL amber bottles, covered with aluminum film, and stored at 4 °C for 21 days. At pre-established time intervals (0, 3, 7, 14, and 21 days), samples were taken, and the microbiological stability of the juice was evaluated by analyzing the native microbiota. Additionally, the quality parameters pH, soluble solids, total polyphenols, and color were measured according to the procedures detailed in Section 2.3. The entire study was performed in triplicate.

#### Microbiological Stability

The following groups of microorganisms were enumerated during storage at 4 °C by the plate count method, following the procedure already mentioned in Section 2.4.5. To determine the Total mesophilic aerobes (TMA), 0.1 mL of the sample (DL: 10) or the corresponding dilution (DL: 100) was spread on PCA and incubated at 37 °C for 48 h. Mold and yeast sample (0.1 mL (DL: 10), or the corresponding dilution (DL: 100)) was spread on the surface of YGC agar and incubated for 5 days at 25 °C. Coliforms and *E. coli* count (1 mL of the corresponding dilution (DL: 10)) was plated on the corresponding Petrifilm 3M^TM^ plate and incubated at 37 °C for 24 and 48 h, respectively. The microbiological criteria to determine the shelf life of the juice were the growth of the native microbiota according to the limits established in the Chilean legislation for pasteurized juices, for which TMA must not exceed 10^3^ CFU/mL, and the absence of *E. coli* [31]. The limit for molds and yeasts was <10^5^ [32].

### 2.6. Statistical Analysis

All experimental data were expressed as mean ± standard deviation (mean ± SD, *n* = 3). To evaluate the differences in the preservation of bioactive compounds and quality parameters among the grape juice treatments and the control, a two-way analysis of variance (ANOVA) was used with a confidence level of 95%. The Tukey test was used to determine significant differences among the samples. Statgraphics Centurion 19 (StatPoint Technologies Inc., Warrenton, VA, USA) was used for all statistical analyses.

## 3. Results and Discussion

### 3.1. Characterization of Grape Juice

The characterization of the physical and chemical parameters of the grape juice is presented in Table 1. The juice had a pH of 3.7, within the pH range of high-acidity juices (2.0–3.7) [33]. Regarding soluble solids, the sample had 25 ± 0.7 °Brix, and the percentage of titratable acidity was 0.6 ± 0.01%. However, Scharf & Sandmann [2] reported a pH of 3.4, and lower content of soluble solids (17.9 ± 0.5 °Brix) for the grape juice cv Thompson Seedless.

The total phenol content of the juice was approximately 214 mg GAE/L. However, Scharf & Sandmann [2] reported a value of 306.2 mg GAE/L in Thompson Seedless grape juice. The antioxidant capacity measured by ORAC was 4.7 mM TE/L, a similar value to that reported in a comparative study of antioxidant capacity in white grape wines, which was found in a range of 4.4–6.1 mM TE/L [34]. However, these values are lower than those reported for red grapes and wines, which can exceed 30 mM TE/L [34]. The antioxidant capacity measured by DPPH· resulted in 17.8 mM TE/L, while a comparative study among red grape juices reported an antioxidant capacity between 11.5 and 54.6 mM TE/L [5].

Regarding physical properties, since it is a cloudy juice, the absorption coefficient (α) was 44.2 cm^−1^, a similar value to that reported for a mixture of Thompson Seedless and Sublima Seedless grape juice (43.4 cm^−1^, 5853 NTU) [35]. The absorption coefficient describes the intensity of attenuation of the light passing through a material. This coefficient of liquid foods is critical for effective UV-C treatment because it affects the microbial inactivation rate. Therefore, when the value of α increases, a decrease is generated in the penetration capacity of UV-C light in the food. In this sense, a high value of α will significantly reduce the antimicrobial efficiency of the treatment at any UV-C dose [36].

Concerning the color of the juice, it presented a clarity (L*) of 53.2. The a* value (red/green coordinates) was −5.0, indicating a green component. The b* value (yellow/blue coordinates) was 28.6 due to a yellow component in the juice sample. These values may be due to the presence of chlorophyll, which is found as chlorophyll a (bluish-green) and chlorophyll b (yellowish-green), the major green pigments in plants, and some other pigments, such as beta-carotene and lutein [37].

The intrinsic UV-absorbing food components, dissolved and suspended solids, turbidity, particle size, and color are critical factors limiting the availability of UV light photons in the bulk of the treated liquid and the consequent UV-C dose [38]. Colored components, solids in suspension, and soluble compounds can reduce the number of photons available to kill microorganisms by absorbing, reflecting, and scattering incident light. They also may provide a site for the aggregation of bacteria [39].

### 3.2. Selection of UV-C Processing Time

**Figure 1:**
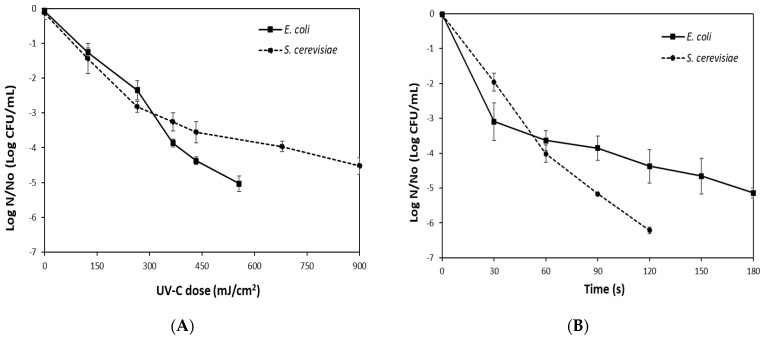
Survival curve of *E. coli* and *S. cerevisiae* in grape juice as treated by (**A**) UV-C light treatment; (**B**) mild thermal treatment at 75 °C. Bars indicate standard deviation.

Figure 1A shows the average survival curve of *E. coli* inoculated into grape juice after processing with UV-C light and the dose received each time. This microorganism was defined as the target microorganism because it is the surrogate of a pathogen that has been associated with outbreaks in fruit juices, so the processing time necessary to reduce it by five logarithmic cycles was determined. A reduction of 5.03 logarithmic cycles of *E. coli* was observed at a 569 mJ/cm^2^ dose of UV-C treatment (12.5 min). Other authors reported a similar degree of inactivation of this microorganism in grape juice by UV-C application. In this sense, Unluturk & Atilgan [39] reported achieving a reduction of 5.34 log cycles of *E. coli* K-12 in a turbid Thompson Seedless grape juice by circulating the juice eight times in an annular flow UV reactor (9900 mJ/cm^2^ at 0.9 mL/s). Similarly, Ramesh et al. [40] demonstrated a reduction of 5 log cycles of *E. coli* ATCC 25,922 in commercial white grape juice by applying UV-C (19.7 mJ/cm^2^).

The presence of yeasts is a frequent cause of spoilage in fruit juices and causes considerable economic losses. Therefore, the effect of UV-C light on *S. cerevisiae* was also evaluated. Figure 1A shows that the inactivation of the yeast (4.36 log CFU/mL) was achieved with a UV-C dose of 900 mJ/cm^2^ applied in 20 min. A higher reduction of up to 5.47 log cycles of *S. cerevisiae* has been reported in a clear Thompson Seedless grape juice by applying a UV-C dose of 136.08 mJ/cm^2^ in 9 min [41]. However, another study that used a cloudy mixture of orange and mandarin juice reached 4.2 log reductions of *S. cerevisiae* by applying a maximum UV-C dose of 1720 mJ/cm^2^ in 15 min [42].

Numerous studies have shown that the intrinsic properties of liquids can significantly influence the antimicrobial effects of UV radiation [15,41,42]. Substances such as sugars, dissolved organic acids, and insoluble suspended solids that increase turbidity result in a higher absorption coefficient that reduces the efficacy of UV radiation by either absorbing or scattering UV light. Additionally, they can provide sites for microorganism adhesion and agglutination [15]. When UV-C light is applied to turbid juices, a higher dose is required to achieve the same inactivation. In this sense, Gouma et al. [43] reported that the UV–C inactivation of *S. cerevisiae* STCC 1172 was independent of pH in the range of 3.0 to 7.0, achieving the same level of inactivation at all assessed pH and UV–C doses. However, the lethality was strongly affected by the absorption coefficient, demonstrating that a decrease in the absorption coefficient increased the UV-C inactivation. They suggested that the effect of the absorption coefficient on UV-C inactivation was due to a physical effect, related to the number of photons interacting with the nucleic acids.

Comparing the effectiveness of UV-C on *E. coli* and *S. cerevisiae*, it was observed that the bacterium was significantly more sensitive to the treatment, exceeding five log reductions at 569 mJ/cm^2^ UV-C dose. However, a significant reduction of yeast was also achieved at a higher UV-C dose (900 mJ/cm^2^). These results agree with other studies regarding the stronger resistance to UV-C irradiation presented by *S. cerevisiae* and other yeasts compared to *E. coli* and other enterobacteria in fruit juices [1,42].

Moreover, *S. cerevisiae* has been mentioned as the most resistant yeast to UV-C light among various spoilage yeasts, followed by *Zygosaccharomyces bailii*, and *Dekkera* spp. [43]. In this sense, Casco et al. [44] reported that UV-C treatment (1271 mJ/cm^2^) caused reductions of 4.4–4.6 log cycles of strain cocktails of *E. coli* and *Salmonella* while reducing only 3.2 log cycles of a yeast mixture in apple juice (pH: 3.97 ± 0.01; α: 0.2 cm^−1^; turbidity: 1115 ± 28 NTU; 12 ± 0.1 °Brix). Yeasts are more UV-resistant than bacteria due to morphological, structural, and compositional differences. The larger size of yeasts compared to bacteria, the higher thickness of their cell wall, the arrangement of DNA inside a nucleus surrounded by histone proteins and the nuclear membrane, and the lower content of thymine or cytosine bases in their genome, among other factors, make it more difficult for UV-C light photons to reach the genetic material and influence the different sensitivity of the eukaryotic yeast, when compared to the prokaryotic bacteria [15,35,43].

Regarding the trend of the curves, *E. coli* followed a negative log-linear correlation between microorganism concentration and UV dose, while the yeast presented a clear non-linear trend in the final stage of inactivation kinetics. Some studies agree that UV-C inactivation of *E. coli* O157: H7, and other bacteria such as *S. aureus*, *S.* Enteritidis, and *L. plantarum*, followed first-order kinetics [45]. Regarding yeasts, the cause of the tailing effect is still a matter of debate, but it could be attributed to several factors, such as a resistant subpopulation, the aggregation of microorganisms, a lack of homogeneity in lighting, the shadowing effect due to the properties of the medium that prevent the passage of UV light, among others [15]. In this sense, Antonio-Gutiérrez et al. [46] studied the effect of UV-C reactors operating in continuous recirculation mode to inactivate *S. cerevisiae* in grape juice and demonstrated that the shape of the curve depended not only on the microorganism, but also on the process conditions that affect its effectiveness. In this sense, the most influential factor was the thickness of the annular gap thickness of the annular gap in the reactor through which the juice passes during the process, and the flow rate of the juice that recirculates in the lamps connected in series.

### 3.3. Selection of the Processing Time for the Mild Thermal Treatment at 75 °C

The impact of mild heat treatment at 75 °C on microorganisms inoculated into grape juice is illustrated in Figure 1. The survival curve of *E. coli* (Figure 1A) indicates that a reduction of 5.14 log CFU/mL was achieved after 180 s of heat treatment. Thus, this time was selected for this thermal process. The bacteria responded to heat treatment with a rapid initial decline, followed by a more resistant subpopulation that declined at a slower rate over time. This resulted in a biphasic curve characterized by two inactivation rates: a faster initial rate and a slower second rate. In the case of *S. cerevisiae*, an inactivation of 5.17 log CFU/mL was achieved after 90 s of treatment at 75 °C (Figure 1B), showing a log-linear inactivation curve. This demonstrates the greater sensitivity of *S. cerevisiae* to heat treatment since it was consistently inactivated in half the time of the bacterium.

Heat exposure triggers a series of cellular events that eventually can lead to the inactivation of microbial cells. Increased membrane permeability can result in the loss of membrane functions, loss of homeostasis, and loss of cellular material. Protein denaturation and aggregation can lead to the loss of specific functions and decrease cell repair capacity. Moreover, alterations in DNA can increase the frequency of mutations [47]. However, the sublethal damage of cells in different proportions of the population and their recovery depends on multiple factors such as the intensity of the thermal treatment applied, the treatment medium, the type of microorganism, and the conditions for recovery [47].

The different responses of bacteria and yeast to heat treatment and the degree of heat resistance vary widely among different microbial groups, due to their differing structure and composition, as well as the mechanisms of resistance they are able to develop. In addition, the composition, physical, and chemical characteristics of the heating media affect microbial inactivation during heat treatment. Sugars have been reported to reduce the thermal inactivation of microorganisms, exerting a protective effect [48]. The presence of divalent cations (especially magnesium ions) may increase heat resistance because it favors the stability of the ribosomes, which is essential for the maintenance of the subunits bound to each other [47]. This might explain the differing behaviors of the observed survival curves. In the case of *E. coli*, some individuals in the population may have experienced sublethal damage in varying proportions, allowing them to survive longer due to several factors. These factors include the intensity of the thermal treatment applied, the high sugar content and mineral levels in the grape juice, and the presence of suspended solids in the pulp, which may have provided some protection to the bacteria. Moreover, yeast cells are generally considered to be more heat-sensitive than vegetative bacterial cells [47].

### 3.4. Effect of UV-C Light and Mild Heat Treatment on Total Phenol Content and Antioxidant Activity

**Figure 2:**
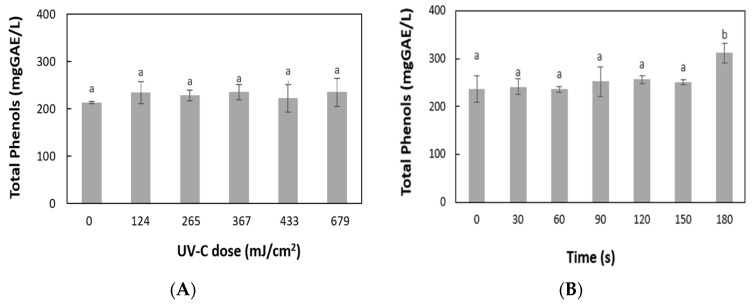
Total polyphenol concentration in grape juice during processing. (**A**) UV-C light treatment; (**B**) mild thermal treatment at 75 °C. Bars indicate standard deviation. Different letters indicate significant differences among treatment times (*p* < 0.05).

Figure 2 shows the variation in total phenol concentration of grape juice during UV-C treatment (Figure 2A) compared to heat treatment (Figure 2B). The sample had an initial total phenol concentration of 214 mg GAE/L and reached a final concentration of 235 mg GAE/L at 679 mJ/cm^2^ UV-C dose. However, the TP content variation was not significant (*p* < 0.05) within that specific UV-C dose range. The effects of UV-C light on total phenols are primarily influenced by the type of matrix exposed to this treatment. In the case of matrices with complex phenolic composition, containing polymers such as condensed tannins, UV-C light would produce some degree of degradation of these polymers, releasing simple phenols, which would induce a slight increase in total phenols. However, this increase would not be significant over a short period of exposure to UV-C light, keeping the TP content relatively stable.

These results are in agreement with those of Hernández-Carranza et al. [21], Caminiti et al. [22], and Ochoa-Velasco & Guerrero Beltrán [23], who did not observe significant changes in the TP of carrot, apple, and red pitaya juices during processing with UV-C light. However, Falguera et al. [19] obtained an increase in phenolic content in grape juices, inferring that UV-C light causes the decomposition of complex phenolic polymers, leading to the release of simpler compounds, which react with the Folin–Ciocalteau reagent, resulting in a higher concentration of total phenols.

In contrast, during heat treatment at 75 °C, the TP increased significantly by thermal action over time from 237 to 312 mg GAE/L at 180 s (Figure 2B). These results agree with those of Genova et al. [49], who report a significant increase in total polyphenols in a Sangiovese grape juice processed at 78 °C for 30 min. This may be because the applied heat can promote the release of phenolic compounds from complex molecules caused by the thermal degradation of membranes and the denaturation of proteins [50,51]. Also, the increase in total phenols (TP) can occur when larger polymers, such as flavan-3-ols derived from condensed tannins, break down. This process exposes more hydroxyl groups that can react with the Folin–Ciocalteau reagent. Additionally, these compounds may remain in unfiltered juices [49].

**Figure 3:**
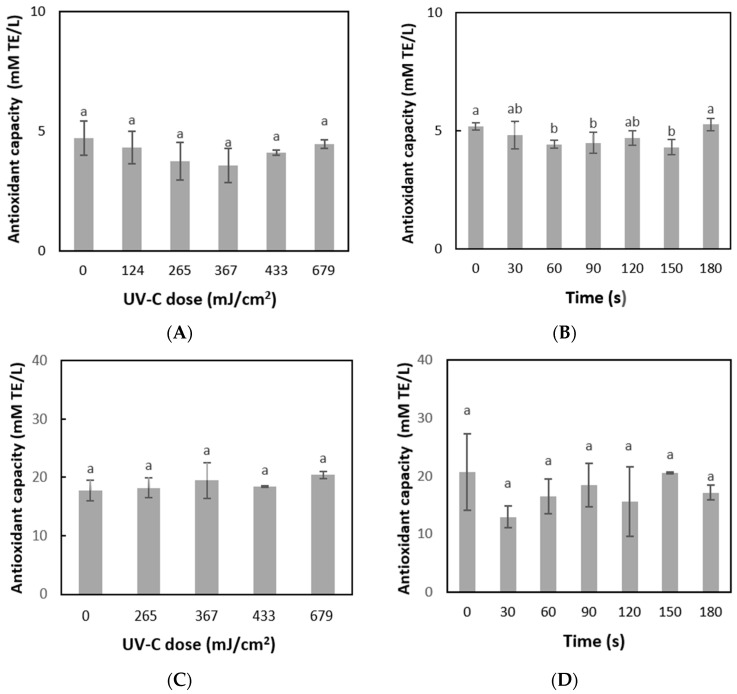
Variation of antioxidant capacity over UV-C dose/processing time in grape juice: (**A**) UV-C treatment measured by ORAC; (**B**) mild thermal treatment at 75 °C measured by ORAC; (**C**) UV-C treatment measured by DPPH; (**D**) mild thermal processing at 75 °C measured by DPPH. Different letters indicate significant differences among treatment times/UV-C dose (*p* < 0.05). Bars indicate standard deviation.

The antioxidant capacity of the juice during UV-C processing and heat treatment was measured through the ORAC (Figure 3A,B) and DPPH^·^ (Figure 3C,D) reactions. The antioxidant capacity of the juice during UV-C processing did not present significant differences (*p* < 0.05), going from 4.7 to 4.5 mM TE/L (ORAC) and from 17.8 to 20.4 mM TE/L (DPPH·) at 12.5 min of treatment. This result coincides with the results obtained by Biancaniello et al. [52], where UV-C light application on cold-pressed green juices in a semi-industrial system was shown to have no influence on the antioxidant capacity measured by ORAC. On the other hand, Souza et al. [53] analyzed the behavior of various cold-pressed juices (lemonade, citrus juices, and green juice) against UV-C in a continuous flow, which showed retention of the antioxidant capacity measured by ORAC.

Regarding the antioxidant capacity of the juice during mild heat treatment (75 °C for 180 s), although significant variation (*p* < 0.05) was observed in the antioxidant capacity for some treatment times, no differences were observed between time 0 and the end of the process (t: 180 s). The antioxidant capacity values remained relatively stable over 180 s at 75 °C, in mild thermal treatment, with around 5.2 mM TE/L (ORAC) and between 20.6 and 17.1 mM TE/L (DPPH·). Similar results were reported by Pala & Toklucu [20] who applied a treatment at 85 °C for 35 min in red and white grape juices without significantly affecting the antioxidant capacity measured by the ABTS radical scavenging activity. The antioxidant capacity did not increase despite increased phenolics because although more phenols were released from complex structures, some of them may have been degraded by the thermal treatment.

The antioxidant capacity results were consistent across the DPPH^·^ and ORAC methods. The DPPH^·^ technique employs an external radical, similar to ABTS, and relies on the transfer of electrons. In contrast, ORAC is based on the transfer of hydrogen atoms, which gives it biological significance. Therefore, it is essential to use various analytical methods to assess antioxidant compounds to capture a broader range of oxidizing species in the samples. Therefore, the results showed that although heat treatment increased the total phenol content, this was not accompanied by an increase in the antioxidant capacity of the juice.

### 3.5. Juice Stability in Refrigerated Storage

Physical and chemical analyses such as pH, soluble solids content, total phenols behavior, and color, as well as the evolution of the microbiota, were monitored to study the stability of grape juice processed by both UV-C and heat treatment, stored at 4 °C for 21 days.

#### 3.5.1. Evolution of pH, Soluble Solids, and Color

The results of pH, soluble solids (°Brix), and color parameters over storage time are shown in Table 2. The pH remained between 3.7 and 3.9, and the soluble solids between 25.6 and 20 °Brix. The pH and the soluble solids did not show significant differences (*p* < 0.05) between the treatments and the control. In addition, the pH remained stable over time, while the soluble solids decreased from day 14 to the end of storage on all the treatments. This decrease may have occurred because the sugar available in the grape juice was the substrate used by the fungi, yeasts, and bacteria that make up the native microbiota of the grape juice.

In agreement with these results, Unluturk & Atilgan [39] observed a significant decrease in the soluble solids content (°Brix) of a cloudy juice of white seedless Sultana grape (pH 3.95; 18.6 °Brix) treated with UV-C light (9.92 J/cm^2^, 0.9 mL/s, 23.5 min) and stored at 4 °C for 14 days. The authors found a decrease in soluble solids of 18% in the control, 20% in the pasteurized juice, and 12% in the UV-C-treated juice during storage time. In contrast, Chang et al. [14] processed white grape juice (pH = 3.52; 16.4 °Brix) at 90 °C for 60 s and evaluated its stability for 20 days at 4 °C. The authors reported that the untreated control and the pasteurized juice showed no significant differences in soluble solids over time. However, in the control, they observed a decrease in pH over time.

The color parameters of the thermally processed, UV-C, and control grape juice are presented in Table 2. The initial clarity (L_ab_*) of the juice was highest for the untreated control, followed by the thermal treatment, and to a lesser extent, for the UV-C treated juice. However, from day 4 onwards, a significant change (*p* < 0.05) in clarity was observed in all treatments and the control, with a lower loss of clarity by the pasteurized juice compared to the control and the UV-C-treated juice. For the rest of the storage time, the juice remained without significant variation until day 21.

Regarding the red-green coordinate (a*), where the positive value indicates the presence of a red component and the negative value indicates a green component, it was observed that initially, all treatments had a green component, which was more remarkable for the control and the UV-C treatment. However, on the fourth day of storage, there was a significant change (*p* < 0.05) for the juice treated with UV-C and the control, which went from a negative value (green) to a positive one (red), remaining this way for the rest of the storage time. The loss of the green component could be due to the degradation of a part of the chlorophyll that gives the characteristic green color to grape juice, which was lost more noticeably in the sample treated with UV-C light, followed by the control. The red component increased due to browning reactions that can occur either by enzymatic or non-enzymatic activity. The thermally processed juice remained almost unchanged throughout the storage, maintaining the green component (a* ≈ −0.3). This may be due to the inactivation of enzymes such as polyphenol oxidase during heat treatment, which prevents browning.

On the other hand, the parameter b* (the positive coordinate indicates a yellow component and the negative one a blue component) indicated that all the juices presented a yellow component, which was higher for the control and heat-treated juice. This component remained without significant variations throughout the storage time. Flavonols, phenolic compounds present in white grapes, have been implicated as responsible for the yellow color of the juice. These compounds would remain stable during storage [54].

Finally, the total color variation (∆*E*) reveals that all treatments significantly changed their color from day 4 onwards, remaining unchanged until the end of storage. Notably, the mild heat treatment exhibited the least color variation (Δ*E* < 3) during storage at 4 °C, showing significant differences compared to the UV-C and control treatments.

Therefore, the color variations were mainly due to the enzymatic action of polyphenol oxidase (PPO), which catalyzes the oxidation of several phenolic substrates and whose polymerization leads to the formation of brown pigments, which can remain residual when the juice is treated with UV-C light. PPO proved to be unstable at temperatures above 40 °C, becoming completely inactive with treatment at 70 °C for 10 min [37]. This is why the lowest total color variation occurred in the juice with the mild heat treatment, probably due to the inactivation of the PPO enzyme, whose residual action was evident in the juice treated with UV-C light and the untreated control, producing the color changes observed during storage. Additionally, the green component associated with chlorophyll may have decreased due to the action of UV-C light since chlorophyll and its precursors are highly photosensitive and can be degraded, causing color changes in the juice [55]. It has also been reported that in acidic conditions, magnesium in the porphyrin ring of chlorophyll tends to be replaced by hydrogen ions, causing a color change from bright green to olive brown, which is called a pheophytinization reaction [37]. Color is a very important attribute for consumers when deciding whether to purchase juice. Alternatives should be considered to prevent color changes in UV-C-processed grape juice. One such option could be the use of natural anti-browning agents such as ascorbic acid, citric acid, etc.

#### 3.5.2. Evolution of Total Phenols During Storage

**Figure 4:**
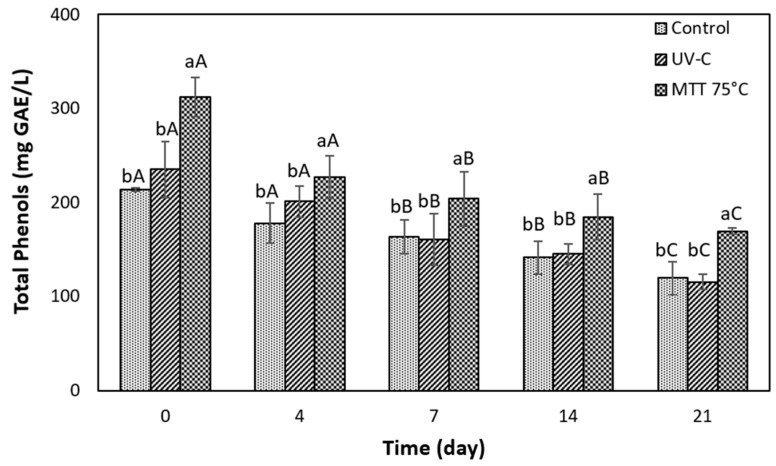
Evolution of the total phenols content of grape juice processed by UV-C or mild thermal treatment (MTT) during storage at 4 °C for 21 days. Different lowercase letters represent statistically significant differences between treatments (*p* < 0.05). Different uppercase letters indicate statistically significant differences between storage times (*p* < 0.05). Bars in the graph indicate standard deviation.

The variation of total phenols during storage is illustrated in Figure 4. It shows that mild thermal treatment from the beginning (t = 0) showed significantly higher TP concentration (25% more) than the control and the juice treated with UV-C light. This difference persisted throughout the storage period and is attributed to the increase in phenolic content achieved through the mild thermal treatment, as discussed in the previous section (Section 3.4). It is important to note that the grape juice processed with UV-C treatment did not show significant differences from the control throughout the evaluation period. This suggests that UV-C processing did not affect the TP content compared to the untreated juice. Similar findings were reported by Gök [56], who investigated the behavior of TP in freshly squeezed grape juice (pH = 4.2; 17.5 °Brix) subjected to UV-C treatment (1232 mJ/cm^2^). The author found that the phenolic content in the untreated and UV-C-treated juices did not differ significantly, exhibiting values close to 350 mg GAE/L.

During storage time at 4 °C, a constant downward evolution of TP concentration was observed for all treatments and the control, i.e., the heat-treated juice started with 312 and ended with 170 mg GAE/L, and the UV-C processed juice started with 235 and ended with 115 mg GAE/L. The decrease in TP was significant from day 7 of storage (*p* < 0.05) and was further accentuated on day 21, with a total reduction of 44% for the control, 51% for the UV-C treated juice, and 46% for the heat-treated juice (Figure 4). The decrease in total phenols of the grape juice during storage occurs due to the partial inactivation of the enzyme polyphenoloxidase. Polyphenoloxidase is responsible for the enzymatic browning that oxidizes phenols to quinones, which polymerize, giving the juice a brown color [57]. This behavior correlates with the increase in the a* parameter in the color measurement. In this sense, Lo’ay et al. [58] observed a decrease of approximately 27% of the TP content in Thompson Seedless grapes on day 20 under refrigeration at 4 °C, finally losing 76% of its phenolic content on day 70 (control), showing a clear tendency towards loss of TP in grape juice over time. Likewise, Ochoa-Velasco & Guerrero Beltrán [23] reported a decrease in total phenols during the storage time in the UV-C light-treated and untreated pitaya juice.

#### 3.5.3. Microbiological Stability of Grape Juice During Refrigerated Storage

**Figure 5:**
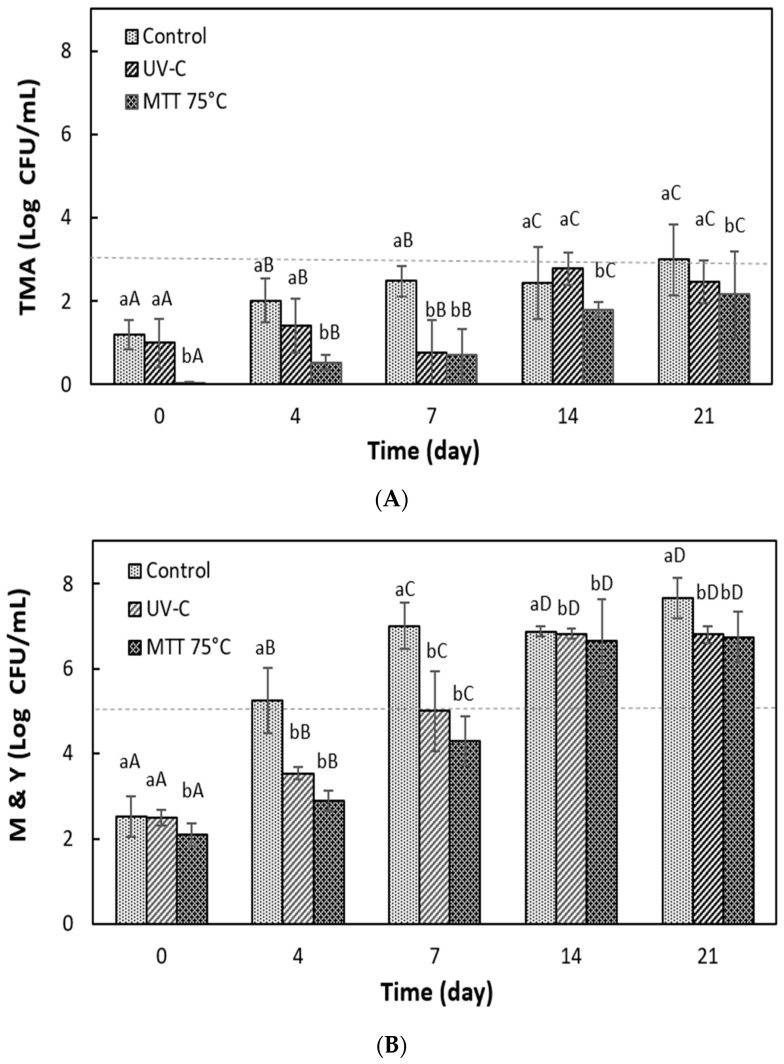
Evolution of the native microbiota of grape juice processed by UV-C or mild thermal treatment (MTT) during storage at 4 °C for 21 days. (**A**) Total mesophilic aerobes (TMA); (**B**) molds and yeasts (M and Y). Different lowercase letters represent statistically significant differences between treatments (*p* < 0.05). Different uppercase letters indicate statistically significant differences between storage times (*p* < 0.05). Bars in the graph indicate standard deviation. (---) Acceptable limit.

The evolution of the grape juice microbiota during refrigerated storage is illustrated in Figure 5. Initially, UV-C treatment was less effective than heat treatment, as the latter achieved complete inactivation of the total mesophilic aerobic count. In contrast, a small viable microbial load remained in the juice treated with UV-C (Figure 5A). During storage, the bacterial load gradually increased. However, because of the intrinsic properties of the juice and the low storage temperature, microbial growth was slowed down. As a result, the TMA counts in both the UV-C and heat-treated juices remained within the acceptable range throughout storage. Conversely, the control exceeded the permissible limit by day 21.

During storage, the bacterial load slowly increased as the intrinsic properties of the juice and the low temperature slowed growth. This, together with the treatments applied, helped the TMA counts achieved by the UV-C and heat-treated juices to remain within the acceptable range during all storage, while the control exceeded the permitted limit on day 21.

In contrast, mold and yeast counts started higher than those of TMA (between 2.1 and 2.5 log CFU/mL) (Figure 5B). These microorganisms found more favorable conditions for their growth, exceeding the limit of 5 log CFU/mL at day 4 in the control. The juices treated with UV-C and MTT remained within the acceptable range until day 7, showing a difference of 2.0 and 2.7 log CFU/mL from the control. This demonstrated the effectiveness of the UV-C and MTT treatments that managed to extend the microbiological stability of the juice by more than 42% (Figure 5B). However, another study reported a higher shelf life of cloudy white grape juice treated by UV-C (9920 mJ/cm^2^) [39]. Initially, the untreated control juice contained 1.35 log CFU/mL of yeast and 1.08 log CFU/mL of LAB. In contrast, the UV-C-treated juice showed no growth of any microorganisms during the first 5 days, after which yeast dominated microbial growth. The UV-C-treated samples reached the yeast spoilage limit after 14 days of storage. Therefore, UV treatment doubled the microbial shelf life compared to the control juice.

No growth of total coliforms or *E. coli* was detected in any of the treated samples or the control during storage at 4 °C. The absence of coliforms confirms the hygienic efficacy of juice processing. Overall, these results align with findings in the literature, which indicate that the maximum microbiological stability of grape juice treated with UV-C light ranges from 7 to 14 days [32,35].

## 4. Conclusions

From a microbiological perspective, kinetic studies have shown that UV-C treatment was more effective than the mild thermal treatment (75 °C for 180 s) in inactivating *E. coli,* achieving the 5-logarithmic cycle reduction required for treating acidic juices. However, UV-C was less effective than MTT in inactivating spoilage yeasts such as *S. cerevisiae*, requiring a higher dose for their inactivation. This is an important consideration, since yeasts play a key role in the microbiological stability of juices during the shelf life.

Furthermore, this technology did not compromise the phenolic content or antioxidant capacity of the grape juice, resulting in a promising alternative to cold pasteurization that preserves these health-promoting compounds. However, this work had some limitations, such as the fact that only one grape variety was analyzed, the enzymatic activity of the treated juices was not determined, and sensory evaluation during storage was not conducted. Therefore, further research is needed to explore strategies to improve the efficiency of the UV-C reactor by incorporating more UV lamps, optimizing process parameters to limit exposure times, and reducing the impact of UV-C light on juice color, considering the potential incorporation of natural anti-browning agents.

## Figures and Tables

**Table 1:** Characterization of grape juice.

Parameter	Value
pH	3.7 ± 0.1
Soluble solids (°Brix)	25.0 ± 0.7
% Acidity (g tartaric acid/100 mL)	0.60 ± 0.01
α_254 nm_ (cm^−1^)	44.2 ± 1.2
Color	
L*	53.20 ± 0.07
a*	−5.00 ± 0.05
b*	28.6 ± 0.2
Total phenols [mg GAE/L]	213.7 ± 2.1
Antioxidant capacity	
ORAC [mM TE/L]	4.7 ± 0.7
DPPH [mM TE/L]	17.8 ± 1.7

Data are presented as mean ± SD of *n* = 3; α_254 nm_: absorption coefficient.

**Table 2:** Quality parameters for the UV-C and mild thermal-treated juices during storage.

Treatment	Time (Day)	pH	Soluble Solids (°Brix)	Color Parameters			
				L_ab_*	a*	b*	∆*E*
Control	0	3.7 ± 0.1 ^aA^	25.0 ± 0.7 ^aA^	53.1 ± 0.4 ^aA^	−4.2 ± 0.9 ^cA^	28.4 ± 0.6 ^aA^	0.00 ± 0.00 ^bA^
	4	3.78 ± 0.08 ^aA^	25.0 ± 0.7 ^aA^	46 ± 1 ^bB^	3 ± 1 ^bB^	26.0 ± 0.5 ^aA^	10 ± 1 ^aB^
	7	3.8 ± 0.1 ^aA^	24.6 ± 0.6 2 ^aA^	48 ± 1 ^bB^	4.0 ± 0.4 ^bB^	26 ± 1 ^aA^	10.11 ± 0.03 ^aB^
	14	3.83 ± 0.08 ^aA^	22 ± 1 ^aB^	50.1 ± 0.3 ^bAB^	2.4 ± 0.3 ^bB^	26.9 ± 0.9 ^aA^	7.4 ± 0.7 ^aB^
	21	3.9 ± 0.1 ^aA^	20 ± 2 ^aC^	48.2 ± 0.2 ^bB^	4 ± 1 ^bB^	26 ± 1 ^aA^	9.35 ± 0.09 ^aB^
UV-C 569 mJ/cm^2^	0	3.76 ± 0.09 ^aA^	24.6 ± 0.9 ^aA^	50.0 ± 0.6 ^cA^	−2 ± 1 ^bA^	26.6 ± 0.2 ^bA^	4 ± 1 ^aA^
	4	3.77 ± 0.08 ^aA^	24.6 ± 0.7 ^aA^	44.2 ± 0.2 ^cB^	5 ± 1 ^aB^	25.0 ± 0.4 ^bA^	8.9 ± 0.4 ^aB^
	7	3.79 ± 0.09 ^aA^	24.4 ± 0.8 ^aA^	43 ± 1 ^cB^	6.1 ± 0.3 ^aB^	25.0 ± 0.9 ^bA^	11 ± 1 ^aB^
	14	3.79 ± 0.05 ^aA^	23.0 ± 0.4 ^aB^	46 ± 1 ^cAB^	6.1 ± 0.2 ^aB^	24 ± 1 ^bA^	10 ± 2 ^aB^
	21	3.80 ± 0.06 ^aA^	21.6 ± 0.5 ^aC^	45 ± 1 ^cB^	6.1 ± 0.7 ^aB^	24.9 ± 0.7 ^bA^	9 ± 2 ^aB^
Thermal treatment 75 °C, 180 s	0	3.7 ± 0.1 ^aA^	25.6 ± 0.7 ^aA^	52.1 ± 0.1 ^bA^	−0.3 ± 0.2 ^cA^	27 ± 1 ^aA^	4.3 ± 0.7 ^aA^
	4	3.77 ± 0.07 ^aA^	25.4 ± 0.6 ^aA^	53.0 ± 0.6 ^aB^	0.2 ± 0.2 ^cB^	26 ± 1 ^aA^	1.4 ± 0.5 ^bB^
	7	3.79 ± 0.06 ^aA^	25.0 ± 0.1 ^aA^	53.2 ± 0.1 ^aB^	−0.1 ± 0.2 ^cB^	26 ± 2 ^aA^	1.5 ± 0.6 ^bB^
	14	3.81 ± 0.06 ^aA^	23 ± 2 ^aB^	55 ± 1 ^aAB^	−0.2 ± 0.3 ^cB^	27 ± 1 ^aA^	2 ± 1 ^bB^
	21	3.84 ± 0.08 ^aA^	20 ± 2 ^aC^	54.8 ± 0.9 ^aB^	−0.3 ± 0.4 ^cB^	26 ± 1 ^aA^	3 ± 1 ^bB^

Results were presented as mean ± standard deviation (*n* = 3). Values in the same columns with different lowercase letters (a–c) indicate significant differences (*p* ≤ 0.05) between treatments for the same storage time. Values with different uppercase letters (A–C) indicate significant differences (*p* ≤ 0.05) through the different storage times.

## Data Availability

The original contributions presented in the study are included in the article, further inquiries can be directed to the corresponding author.

## Abbreviations

The following abbreviations are used in this manuscript:

UV-C	Short-wave ultraviolet light
MTT	Mild thermal treatment
TP	Total phenols
AC	Antioxidant capacity
GAE	Galic acid equivalent
TE	Trolox equivalent
α	Absorption coefficient
AOAC	Association of the Official Analytical Chemists
DL	Detection limit
LAB	Lactic acid bacteria
